# Dramatic pain relief and resolution of bone inflammation following pamidronate in 9 pediatric patients with persistent chronic recurrent multifocal osteomyelitis (CRMO)

**DOI:** 10.1186/1546-0096-7-2

**Published:** 2009-01-12

**Authors:** Paivi MH Miettunen, Xingchang Wei, Deepak Kaura, Walid Abou Reslan, Alberto Nettel Aguirre, James D Kellner

**Affiliations:** 1 Department of Pediatrics, Alberta Children's Hospital and University of Calgary, 2888 Shaganappi Trail NW, Calgary, Alberta, T3B 6A8, Canada; 2 Division of Pediatric Rheumatology, Alberta Children's Hospital and University of Calgary, 2888 Shaganappi Trail NW, Calgary, Alberta, T3B 6A8, Canada; 3 Department of Diagnostic Imaging, Alberta Children's Hospital and University of Calgary; 4 Community Health Sciences Department, Alberta Children's Hospital and University of Calgary, 2888 Shaganappi Trail NW, Calgary, Alberta, T3B 6A8, Canada; 5 Divison of Pediatric Infectious Diseases, Alberta Children's Hospital and University of Calgary, 2888 Shaganappi Trail NW, Calgary, Alberta, T3B 6A8, Canada

## Abstract

**Background:**

Chronic recurrent multifocal osteomyelitis (CRMO) is an inflammatory, non-infectious osteopathy that affects predominantly patients ≤ 18 years of age. There is no uniformly effective treatment. Our objective is to describe clinical, magnetic resonance imaging (MRI), and bone resorption response to intravenous pamidronate in pediatric CRMO.

**Methods:**

We report our prospectively documented experience with all CRMO patients treated with pamidronate between 2003 and 2008 at a tertiary pediatric centre. Pamidronate was administered as intravenous cycles. The dose of pamidronate varied among subjects but was given as monthly to every 3 monthly cycles depending on the distance the patient lived from the infusion center. Maximum cumulative dose was ≤ 11.5 mg/kg/year. Pamidronate treatment was continued until resolution of MRI documented bone inflammation. Visual analog scale for pain (VAS) and bone resorption marker urine N-telopeptide/urine creatinine (uNTX/uCr) were measured at baseline, preceding each subsequent pamidronate treatment, at final follow-up, and/or at time of MRI confirmed CRMO flare. MRI of the affected site(s) was obtained at baseline, preceding every 2^nd ^treatment, and with suspected CRMO recurrence.

**Results:**

Nine patients (5 F: 4 M) were treated, with a median (range) age at treatment of 12.9 (4.5–16.3) years, and median (range) duration of symptoms of 18 (6–36) months. VAS decreased from 10/10 to 0–3/10 by the end of first 3–day treatment for all patients. The mean (range) time to complete MRI resolution of bone inflammation was 6.0 (2–12) months. The mean (confidence interval (CI)) baseline uNTX/uCr was 738.83 (CI 464.25, 1013.42)nmol/mmol/creatinine and the mean (CI) decrease from baseline to pamidronate discontinuation was 522.17 (CI 299.77, 744.56)nmol/mmol/creatinine. Median (range) of follow-up was 31.4 (24–54) months. Four patients had MRI confirmed CRMO recurrence, which responded to one pamidronate re-treatment. The mean (range) uNTX/uCr change as a monthly rate from the time of pamidronate discontinuation to flare was 9.41 (1.38–19.85)nmol/mmol/creatinine compared to -29.88 (-96.83–2.01)nmol/mmol/creatinine for patients who did not flare by the time of final follow-up.

**Conclusion:**

Pamidronate resulted in resolution of pain and MRI documented inflammation in all patients. No patient flared while his/her uNTX/uCr remained suppressed. We propose that pamidronate is an effective second-line therapy in persistent CRMO.

## Background

Chronic recurrent multifocal osteomyelitis (CRMO) is an auto-inflammatory osteopathy that predominantly affects children [1-3]. It is characterized by bone pain, soft tissue swelling and osteolysis. SAPHO syndrome (synovitis, acne, pustulosis, hyperostosis and osteitis) includes similar bony lesions, and the dermatologic manifestations may be absent in childhood [4]. A recent series suggests that unifocal and/or non-recurrent non-bacterial osteomyelitis may represent different levels of severity within the same clinical spectrum, and a new classification has been proposed to include all of these disorders under the umbrella term of chronic non-bacterial osteomyelitis (CNO) [5]. Within CNO, the existing entity of CRMO represents the more severe end of the spectrum [5]. In this manuscript, the term CRMO is retained to describe this subgroup of CNO patients.

Although majority of patients with CRMO have resolution of symptoms post-pubertally, the bone pain in active disease is severe [6-9]. In addition, long-term studies reveal that up to a quarter of patients have persistent disease, with risk for permanent bony deformities, poorer quality of life, and difficulty in achieving vocational goals [1,2,10,11]. There is no cure, and the goal of management is effective pain control [1,2]. No single agent has proven to be consistently effective, although analgesics, nonsteroidal anti-inflammatory agents (NSAIDs) [5,12], sulfasalazine, methotrexate, corticosteroids and infliximab have all been tried [3]. There is a need to develop new therapies for treatment resistant patients.

Histology [6,12,13] reveals inflammatory changes, and increased osteoclasts and bone resorption characterize early lesions. Plain radiographs of early CRMO document osteolytic lesions with periosteal reaction [9,11-13]. Magnetic resonance imaging (MRI) [14] is a non-invasive imaging modality that is highly sensitive to active and remitted inflammatory lesions in bone and soft tissues in CRMO [14,15]. Active lesions occur with increased signal intensity on short tau inversion recovery (STIR) or fat-saturated T2-weighted images and decreased signal intensity on T1-weighted images, consistent with inflammation [15]. Conversely, resolved CRMO is reflected by no signs of inflammation by MRI [5].

Because bisphosphonates inhibit bone resorption, have pain modifying effect and demonstrate anti-inflammatory action [3], they seemed excellent candidates for use in CRMO. In year 2003, when our first patient was treated, there were several reports of beneficial effect of bisphosphonates in adult patients with inflammatory osteitis, such as symphysitis pubis and ankylosing spondylitis [16,17], and in non-inflammatory pediatric bone disorders, such as osteogenesis imperfecta [18] and polyostotic fibrous dysplasia of the bone [19]. In addition, there was one existing description in an abstract form of successful use of pamidronate for rapid pain relief in a pediatric patient with long-standing CRMO [20]. These encouraging reports prompted us to pursue pamidronate for those patients with persistently active CRMO despite conventional treatment.

Urinary N-telopeptide/urine creatinine (uNTX/uCr) is a marker of collagen-1 breakdown. It is traditionally used to monitor treatment response to bisphosphonates in adult patients with bone diseases characterized by accelerated bone turnover, such as Paget's disease of bone [21,22]. It has not been previously studied in pediatric inflammatory osteopathies.

We present our clinical experience using pamidronate in nine children with CRMO, in whom we prospectively collected data on clinical features, pain, MRI and uNTX/uCr response.

## Methods

### Patients

CRMO was diagnosed in 17 children from 2003 to 2008 at our tertiary care hospital. The diagnosis of CRMO was made based on the following critera: (1) ≥ 2 typical bony lesions (osteolysis with surrounding sclerosis on conventional radiographs); (2) duration ≥ 6 months; (3) typical histologic features (acute/chronic inflammation) on bone biopsy; (4) and age < 18 years at diagnosis [12]. Eight children responded to NSAIDs and were not considered for pamidronate treatment. Sulfasalazine and/or prednisone were offered after NSAID failure. All but one family declined these medications. The reasons for the families' reluctance to proceed with these other medications included: 1) the already significant functional disability of the child; 2) concern for known side effects of especially prednisone; and 3) pre-existing allergy to sulfasalazine. In total, 9 patients continued to have severe pain, functional limitations, and significant school/day-care absences despite treatment with NSAIDs, analgesics, opioids, and/or sulfasalzine and prednisone, and were offered pamidronate (Table 1).

**Table 1:** Baseline characteristics of study patients with CRMO.

Total number of patients	9
Sex (F:M)	5:4

Age at pamidronate treatment (y)*	12.9 (4.5–16.3)

Duration of symptoms before IVP (m)*	18 (6–36)

HLA-B27 positive (n)	3/9

VAS for pain pre-IVP (scale 0–10)	10/10 for all patients

Functional limitation pre-pamidronate	
*Prolonged school/daycare absences (n)*	9/9
*Hospitalized for pain management (n)*	4/9
*Limited use of upper limb (n)*	3/9
*Difficulty weight bearing (n); requiring crutches (n)*	5/9; 2/9
*Bedridden from spinal/leg pain (n)*	2/9

Extra-osseus manifestations at baseline	
*Psoriasis/palmoplantar pustulosis/acne*	0/9
*Synovitis/inflammatory bowel disease*	0/9

Number of patients with elevated ESR at baseline	6/9
ESR value (range) at baseline (normal < 10 mm/Hg)*	23.7 (3–52 mm/Hg)

Bone involvement pre-IVP as documented by MRI	
Number of affected sites*	3.5 (2–9)
*Clavicle (n)*	2/9
*Sacroiliac joint region (n)*	2/9
*Distal fibula (n)*	2/9
*Distal femur (n)*	4/9
*Vertebrae (n; range for number of involved vertebrae)*	2/9 (3–9)
*Distal tibia (n)*	2/9
*Proximal humerus (n)*	1/9
*Distal radius (n)*	1/9

Clinically evident adjacent soft tissue swelling pre-pamidronate	
*Number of patients*	5/9

Bone histopathology	
Number of patients with bone biopsies** (n)	9/9
*Active inflammation only (n)*	5/9
*Chronic inflammation only (n)*	1/9
*Active and chronic inflammation (n)*	3/9
*Increased bone resorption and osteoclasts (n)*	1/9
*Necrotic bone (n)*	2/9
*Reactive new bone (n)*	1/9

Previous treatment pre-IVP	
Nonsteroidal anti-inflammatory drugs (n)***	9/9
*Sulfasalazine (50 mg/kg/day) (n)*	1/9
*Prednisone (oral, 10 mg/day) (n)*	1/9
*Codeine (n)*	9/9
*Fentanyl (n)*	3/9
*Morphine (n)*	1/9
*Ketorolac (n)*	1/9
*Amitriptyline (n)*	1/9
*Surgical courettage (n)*	2/9

CRMO = chronic recurrent multifocal osteomyelitis.

*Data is represented as median (range).

** The tissues derived from these biopsies were fixed in neutral-buffered formalin, decalcified with 8% formic acid, and processed routinely with paraffin embedding. These biopsies were performed at the initial presentation with bone pain to rule out infection/malignancy.

*** Nonsteroidal anti-inflammatory drugs included the following: naproxen sodium [up to 15 mg/kg/day], celecoxib [up to 200 mg/day], ibuprofen [up to 10 mg/kg/dose 4 times daily]

y = years.

m = months.

VAS = visual analogue scale for pain.

n = number of patients.

ESR = erythrocyte sedimentation rate.

IVP = intravenous pamidronate.

### Treatment

Pamidronate was administered as intravenous cycles. Patients received 1 mg/kg/day (maximum 60 mg/day) for all but the very first infusion, which was 0.5 mg/kg/day. The first 2 male patients received a 3-day treatment once monthly. In subsequent patients, the treatment plan consisted of an initial 3-day treatment, followed by either 1-day treatment monthly, or 3-day treatment every 3 months, with a maximum cumulative dose of no more than 11.5 mg/kg/year. The different treatment frequencies were used to accommodate families who had to travel long distances to the hospital. A 1-day pamidronate treatment was repeated (1 mg/kg/day, with maximum dose of 60 mg/day) in cases of MRI-confirmed CRMO flare. It was explained to all families that the treatment was off-label. Patients were counseled for potential side effects, including fever and myalgia, and after year 2006 – when cases of osteonecrosis of the jaw were reported in adult patients who were treated with bisphosponates – for the possibility for osteonecrosis of the jaw [23]. All parents/patients gave consent to treatment with pamidronate. The patients' calcium and Vitamin D intakes were maintained at 800–1000 mg of elemental calcium and 400 IU of Vitamin D daily. NSAIDs were discontinued two weeks prior to pamidronate treatment. NSAID use was permitted in case of suspected CRMO flare.

### Clinical measures

"Pain" was recorded at baseline, at each pamidronate treatment and at potential CRMO recurrence using a 10-cm visual analog scale, anchored by "severe pain" and "no pain". Patients' height was measured at baseline, and at each follow-up.

### Extra-osseous manifestations

Data was collected regarding extra-osseous manifestations that can be seen in association with CRMO, including dermatologic manifestations (acne, palmoplantar pustulosis and psoriasis), synovitis, and inflammatory bowel disease.

### Magnetic resonance imaging (MRI)

Bone and soft tissue inflammation in active CRMO was confirmed by MRI, which was obtained at base-line and prior to every 2^nd ^treatment. MR images were acquired from clinical Siemens 1.5 T scanners (Erlagen, Germany). Multiple spin-echo T1-weighted images, fat-saturated T2-weighted images (T2 FS) and post-gadolinium fat-saturated T1-weighted images were routinely obtained. Short-tau inversion recovery (STIR) images were obtained in some patients instead of T2 FS, and regarded as equivalent to T2 FS images. Once there was over 90% improvement in inflammation, pamidronate was discontinued. In case of suspected flare, MRI was repeated within 4 weeks of symptom recurrence. The improvement or progression of lesions was quantified in lesion volume measured on multi-plane MR images, using either STIR or T2 FS images.

### Laboratory measurements

Bone resorption was assessed by measurement of urinary cross-linked N-telopeptides of type I collagen related to creatinine (uNTX/uCr, in nmol/mmol/creatinine) by enzyme-linked immunoabsorbent assay (Osteomark; Ostex, Seattle, WA, USA) using the second void sample of the morning. Each patient was compared to age and sex specific uNTX/uCr ratios for healthy boys and girls [24]. UNTX/uCr was measured: a) just prior to the first pamidronate treatment; b) on day 3 of the first treatment; c) preceding each subsequent treatment; d) at time of confirmed CRMO flare; or e) at time of final follow-up for those patients who did not flare during the study period. Serum alkaline phosphatase (a marker for bone formation), erythrocyte sedimentation rate (ESR), and complete blood count (CBC) were measured at baseline and at monthly intervals during pamidronate treatment. C-reactive protein (CRP) was not routinely measured. All laboratory tests were repeated at time of suspected CRMO flare. Serum calcium was measured just preceding each infusion, and one hour after completion of infusion to monitor for possible hypocalcemia which can be seen as a side effect of bisphosphonate infusion.

### Histology

All patients had a bone biopsy prior to diagnosis of CRMO to exclude malignancy or infectious osteomyelitis.

## Results

Nine patients (five female and four male) were treated with pamidronate (Tables 1, 2). The median (range) age at pamidronate treatment was 12.9 (4.5–16.3) years, and the median (range) of duration of symptoms was 18 (6–36) months. Median (range) duration of follow-up after the first dose of pamidronate was 31.4 months (24–54) (Table 2).

**Table 2:** Data on pamidronate treatment, MRI response, MRI confirmed CRMO relapse, and follow-up

IVP dosing frequency	
*One day monthly (n)*	6/9
*Three day cycle every 3 months (n)*	3/9
Cumulative dose of pamidronate in mg/kg/year*	5.0 (4.5–9.5)

Time to > 90% MRI signal resolution after initial treatment (m)*	6.0 (2–12)

Number of IVP cycles required for > 90% MRI signal resolution*	5 (2–10)

Number of patients with clinical resolution of adjacent soft tissue swelling	5/5

Side effects from IVP	
*Myalgia and fever with first dose (n)*	4/9
*Osteonecrosis of the jaw (n)*	0/9

Duration of follow-up after first IVP (m)*	31.4 (24–54)

Number of patients who relapsed	4/9

Time from first IVP to relapse (m)*	12.3 (12–18)

Time to >90% MRI signal resolution after repeat IVP treatment for CRMO relapse (m)	2 (for all patients)

Extra-osseus manifestations during follow-up	
*Psoriasis/palmoplantar pustulosis/acne*	0/9; 0/9; 1/9
*Synovitis/inflammatory bowel disease*	0/9; 0/9

*Data presented as median (range).

MRI = magnetic resonance imaging.

IVP = intravenous pamidronate treatment.

n = number of patients.

m = months.

The first male and the first female patients are described in the text, and the rest in the tables and figures.

### Patient 1

This 16-year-old boy had severe bone pain, involving sequentially right clavicle, left sacrum and right sacrum over preceding 36 months. HLA-B27 was negative. A biopsy of the right clavicle showed minimal inflammation. CRMO was diagnosed when he developed MRI documented involvement of the ileum adjacent to each sacroiliac joint (SIJ). Despite prolonged physiotherapy, analgesia, naproxen (15 mg/kg/day), sulfasalazine (2 grams/day), fentanyl, and prednisone (10 mg/day for 5 days, resulting in psychosis) he had incapacitating pain in the right sacrum for 7 months preceding pamidronate treatment. He was unable to bend forward, walk without a limp, run, or sit for the duration of a regular school class.

**Figure 1:**
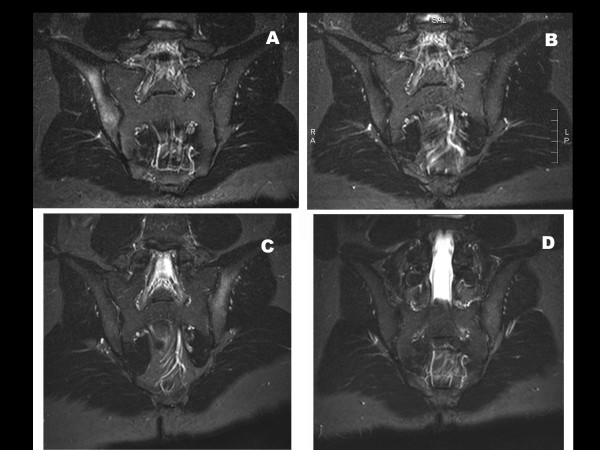
**Imaging data of sacral involvement by CRMO in patient 2, a 16-year-old boy**. **(A) Pre-treatment imaging**: Coronal STIR MRI images reveal increased T2 signal in the ilium adjacent to the right sacroilial joint (SIJ), consistent with inflammation. **(B) Imaging 2 months after initiation of pamidronate**: Abnormal signal on coronal STIR has resolved. **(C) Imaging at clinical relapse**: Coronal STIR MRI image reveals increased T2 signal in the ilium adjacent to the left SIJ, consistent with inflammation. The previously affected right ilium demonstrates no abnormal signal. **(D): Imaging 2 months post 1 day pamidronate retreatment**: Previously demonstrated abnormal signal on coronal STIR is no longer seen.

He received 2 monthly pamidronate treatments. The first dose resulted in complete pain resolution on VAS. MRI of the right sacrum had normalized by 6 weeks (Figure 1). UNTX/uCr decreased from 165 to 28.3 nmol/mmol/creatinine after the first treatment. He required two re-treatments, 18 and 27 months following the first treatment for MRI confirmed flare (Figure 1), with complete clinical and MRI response. At each flare, uNTX/uCr had increased again (170.2 and 120 nmol/mmol/creatinine, respectively). He remains clinically well at 54 month follow-up.

### Patient 2

This 11-year-old girl had severe bone pain affecting sequentially the left and right femurs over a 13-month period, associated with soft tissue swelling. Her initial presentation involved the left femur only, and malignancy or osteomyelitis was suspected. Left femoral biopsy revealed acute inflammation. She was started on parenteral cloxacillin, although her cultures were negative.

**Figure 2:**
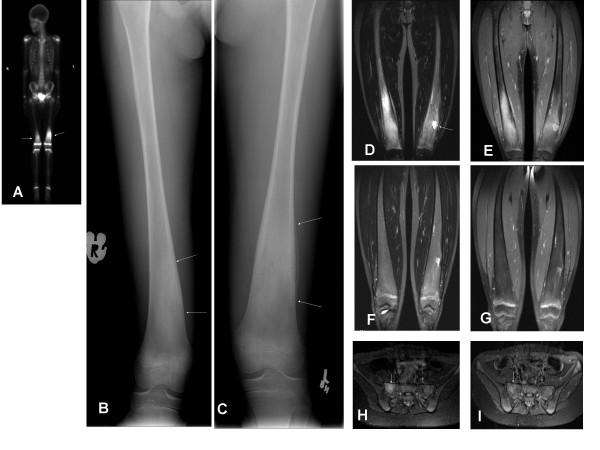
**Imaging data of CRMO involving both distal femurs in patient 2, an 11-year-old girl**. **(A-E) Pre-treatment imaging**: (A) Pre-treatment technetium^99^nuclear bone scan demonstrates abnormal uptake in both distal femurs with more prominent changes on the left (arrow). (B-C) Anterior-posterior radiographs of both distal femurs demonstrate ill-defined areas of sclerosis (arrows) mixed with small focal lucent areas. Periosteal reaction is noted on the left side. (D) Coronal STIR MRI images obtained 4 months after the bone scan reveal increased signal in the right femur. The left femur reveals post-operational changes on the site of previous bone biopsy (arrow). (E) Post-gadolinium fat-saturated T1 weighted image shows marked enhancement of the right distal femoral lesion, with post-operational changes in the left femur. **(F-G) Imaging 10 months after initiation of pamidronate**: Complete resolution of the right femoral lesion is demonstrated on STIR image (F) and post-gadolinium fat-saturated T1 weighted image (G). **(H) Imaging at clinical relapse**: Coronal STIR MRI images reveal increased T2 signal in the right aspect of the sacrum, consistent with inflammation. **(I): Imaging 2 months post 1 day pamidronate retreatment**: Previously demonstrated abnormal signal on coronal STIR has resolved (arrow).

CRMO was diagnosed 7 months later when the right femur became involved despite ongoing treatment with cloxacillin. Pre-pamidronate radiographs revealed sclerosis and osteolytic lesions in both femurs, with periostitis in the left distal femur, corresponding to hot-spots on bone-scan (Figure 2). She was HLA-B27 positive. She was unable to walk unassisted despite a prolonged course of naproxen (15 mg/kg/day) and codeine. The family declined corticosteroids and sulfasalazine.

She received a total of three every 3-monthly pamidronate treatments. VAS for pain resolved completely by day 3 of the first treatment, accompanied by decrease in the soft tissue swelling. MRI of both distal femurs at baseline documented bone inflammation (Figure 2). MRI signal abnormalities normalized by the 3rd treatment, when pamidronate was discontinued (Figure 2). To date, she has required one further course of pamidronate for MRI confirmed new CRMO lesion in the right sacrum at 14 months following the first treatment, with complete pain resolution the day after. The abnormal MRI signal resolved by 2 months following re-treatment (Figure 2).

The uNTX/uCr decreased from 1361.0 to 395.4 nmol/mmol/creatinine by day 3 of first treatment. It had increased to 504.0 nmol/mmol of creatinine at the time of CRMO flare. She remains clinically well at 39-month follow-up.

### Other data

#### Clinical measurements

##### Pain, function, and day-care/school absences (Table 2)

All patients had significant and sustained pain relief following pamidronate, with a decrease in VAS from 10/10 to 0–3/10 after the first treatment. Each patient recovered full function of the areas affected by CRMO, returned to daycare/school within 1 week after the first treatment, and has not had further absences due to CRMO related pain.

A total of 5 patients developed recurrence of pain 12–18 months following the first pamidronate dose, unresponsive to NSAIDs. MRI confirmed CRMO relapse in four patients and revealed non-CRMO etiology for the remaining patient (vertebral compression fracture from spinal CRMO). All four patients responded to one-day pamidronate re-treatment with complete pain resolution.

##### Extra-osseous manifestations

One of the patients developed moderate acne at age 17 years, after completion of pamidronate. No patient developed psoriasis, palmoplantar pustulosis, synovitis, or inflammatory bowel disease during the follow-up period.

##### Magnetic resonance imaging (Table 2, Figures 1, 2, 3, 4)

**Figure 3:**
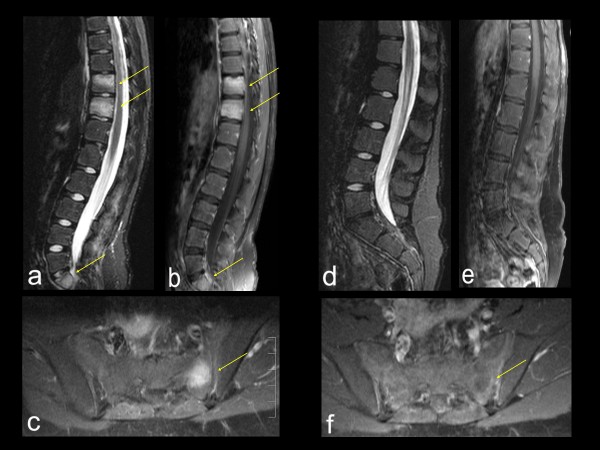
**Imaging data of spinal and sacral CRMO lesions in a 10-year old girl**. **(A-C)**. **Pre-treatment sagital (A and B) and axial (C) MRI**. (A) STIR sequence and (B and C) post-gadolinium T1-weighted sequence reveal abnormal signal in vertebral bodies of T10, T11, and S1 (arrows), as well as in sacral ala (arrow). **(D-F)**. **Post-treatment (5 months after initiation of pamidronate) MRI **using the same technique as (A-C): Complete resolution of the previously seen abnormal signal.

**Figure 4:**
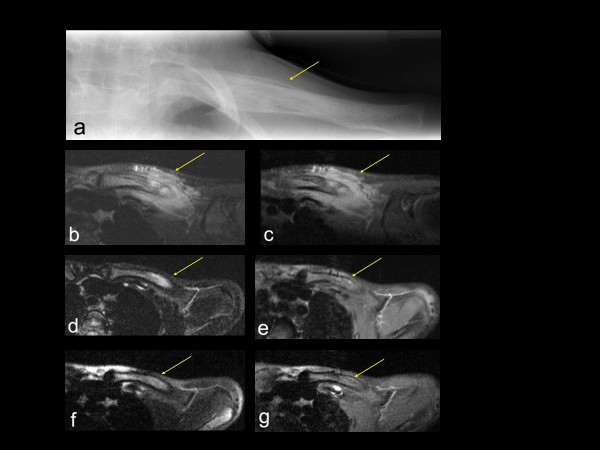
**Imaging data of CRMO lesion involving left clavicle in a 7-year old girl (A). Pre-treatment imaging**. Plain radiograph of the left clavicle demonstrates periosteal new bone formation (arrow). **(B-C)**. **Pre-treatment MRI**: (B) Axial (fat-saturated, T2-weighted) and (C) post gadolinium MRI: Hyper-intense T2 signal with post-contrast enhancement is seen within the clavicle (arrow) with marked soft tissue inflammation (arrow). **(D-E)**. **Post-treatment MRI (5 months after initiation of treatment with pamidronate) **with the same technique as B and C, respectively. The intra-osseous abnormal signal has significantly improved, and marked soft tissue abnormality has almost completely resolved. **(F**-**G)**. **Post-treatment MRI (8 months after initiation of treatment with pamidronate) **with the same technique as B and C, respectively, reveals complete resolution of the intra-osseous abnormal signal.

Base line MRI confirmed inflammatory lesions in all patients, with decreased T1 signal, increased T2 signal, and post-gadolinium enhancement in affected bone and/or soft tissues (Table 2). The abnormal signal improved dramatically after the first treatment, with further improvement with subsequent treatments. The mean time for > 90% resolution of MRI signal abnormality was 6.0 months. The MRI abnormalities following re-treatment for four patients with CRMO flare resolved within 2 months.

In addition to figures 1, 2 which show the effects of pamidronate in our first male and first female patients, figures 3, 4 demonstrate effects of pamidronate on diagnostic imaging of CRMO involving spine, sacrum, and clavicle. Figure 3 depicts MRI appearance of CRMO involving spine and sacral ala in a 10-year old girl at baseline and following pamidronate, revealing eventual resolution of bone marrow edema of all affected sites. Figure 4 demonstrates characteristic radiographic appearance of active CRMO involving a clavicle, and illustrates the sequence of abnormal MRI signal resolution following pamidronate. The base line MRI reveals robust soft tissue inflammation in addition to bone marrow edema, not evident on plain radiograph. The soft tissue edema resolved much more rapidly than the bone marrow edema, but eventually both resolved completely.

#### Laboratory measurements

##### UNTX/uCr (Figure 5)

**Figure 5:**
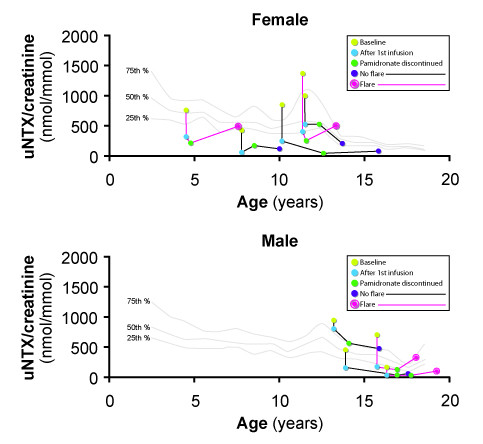
**Urinary N-telopeptide/urinary creatinine ratio (uNTX/uCr) in girls (*upper panel*) and in boys (*lower panel*)**. Data is shown for each individual patient prior to the first intravenous pamidronate treatment (IVP); just after the first IVP; at the time of pamidronate discontinuation; and either at the time of last follow-up for patients who did not flare or at the time of CRMO flare. Continuous lines represent the 75^th ^(*top*), 50^th ^(*middle*), and 25^th ^(*bottom*) percentile, respectively, of the reference range for healthy subjects [24].

Baseline uNTX/uCr values were within the 25^th ^to 75^th ^percentiles of age-expected range for five out of nine patients, and two females and two males had values above the 75^th ^percentile. The mean (confidence interval (CI)) pre-first pamidronate treatment uNTX/uCr was 738.83 (CI 464.25, 1013.42)nmol/mmol/creatinine and the mean (CI) decrease in uNTX/uCr after the first treatment was 440.99 (CI 246.33, 635.65)nmol/mmol/creatinine. The mean (CI) decrease in uNTX/uCr from baseline to discontinuation was 522.17 (CI 299.77, 744.56)nmol/mmol/creatinine. The mean (range) uNTX/uCr change as a monthly rate from the time of pamidronate discontinuation to flare for patients with CRMO relapse was 9.41 (1.38–19.85)nmol/mmol/creatinine per month compared to -29.88 (-96.83–2.01)nmol/mmol/creatinine per month for patients who did not flare by the time of final follow-up.

##### Other laboratory measurements (Table 1)

Apart from mildly elevated ESR at baseline in 6 patients all other laboratory markers were unremarkable. Three patients out of nine were HLA-B27 positive. No patient had hypocalcemia with pamidronate infusions.

##### Histopathology (Table 1)

All pre-treatment bone biopsies demonstrated inflammation, and one demonstrated increased osteoclasts with bone resorption. No patient had a repeat bone biopsy following pamidronate.

##### Linear growth

Growth velocity was maintained at ≥ 5 cm/year for all patients whose epiphyses were not closed by the first pamidronate treatment (data not shown).

##### Secondary effects of pamidronate (Table 1)

Four patients developed fever and myalgia with the first pamidronate dose. No osteonecrosis of the jaw was observed.

## Discussion

We have presented data on clinical and MRI response to pamidronate in nine pediatric patients with persistent CRMO. Dramatic pain relief, followed by an impressive improvement in bone inflammation was documented in all patients. Four children had MRI confirmed relapses 12 – 18 months following pamidronate treatment, with prompt response to re-treatment.

Although the individual course of CRMO can vary, long-term studies emphasize the potentially debilitating nature of this condition. A 2007 review of 39 pediatric CRMO patients by Jansson et al reported a median disease duration of 21 months, with 51% of patients having ongoing active disease in the preceding 18 month period [3]. Other studies have revealed disease duration for up to 14–25 years [1,2,15]. Huber et al reporting on 23 Canadian patients [2] and Catalano-Pons et al reporting on 40 patients from a multi-center study in France[25] confirm that for patients with persistent disease, the adverse effects can extend into future and include: 1) poorer quality of life[2]; 2) risk for bone abnormalities (kyphosis, vertebral fractures, and limb length discrepancy); and 3) potentially worse vocational outcome [1-4,9,25,26]. Even those patients who eventually recover have significant pain during CRMO flares, with adverse effect on general well-being. Although anti-inflammatory medications and surgery are effective for some patients, there is a need to find effective treatment for refractory cases.

Pamidronate was chosen because of beneficial reports in one pediatric patient with CRMO [20] and in several adult patients with inflammatory osteitis [16,17,27]. During our study period, other reports in the literature emerged, confirming the efficacy of bisphosphonates for pain relief in pediatric and adult onset SAPHO syndrome [4,21], in pediatric patients with chronic inflammatory lesions of the mandible [28], and in further cases of CRMO [29,30]. According to the new proposed classification of chronic non-bacterial osteomyelitis (CNO) as an umbrella term for CRMO, SAPHO syndrome, and unifocal/non-recurrent non-bacterial osteomyelitis [5], the equally beneficial effects of bisphosphonates in these disorders seem to be supportive of a shared pathogenesis.

Pathogenesis of CRMO/CNO continues to be poorly understood. It is not known what triggers the initial episode, and why some patients have more persistent disease than others. However, both local and systemic increase of tumor necrosis factor alpha (TNF-α) has been documented in active CRMO/CNO [3]. In keeping with this observation, beneficial effects of anti-TNF agents have been reported in a few patients each with CRMO [31], SAPHO syndrome [32], and inflammatory bowel disease related CRMO [33]. Indeed, some of the anti-inflammatory effects of bisphosphonates are proposed to be secondary to their ability to suppress proinflammatory cytokines, such as TNF-α, interleukin (IL)-6, and IL-1 [16].

The dosing and scheduling regimen for pamidronate in our study was adapted empirically from the schedule used in treating osteogenesis imperfecta [18]. Although we had designed our study to allow for a maximum cumulative pamidronate dose of 11.5 mg/kg/year, no patient required this maximum dose.

The most striking clinical observation was rapid pain relief within 48 hours following the first pamidronate treatment, regardless of the location of CRMO lesions. The effective pain relief following pamidronate has now been observed in a variety of pediatric CNO disorders. The 1999 abstract by Seibel et al was the first to document dramatic pain relief following pamidronate in an adolescent boy with a 6-year history of intermittently active CRMO [20]. This patient received 30 mg of IV pamidronate, with complete pain resolution within 4 days. His radiographs gradually improved over one year, although they did not completely normalize. The beneficial effect of pamidronate in childhood onset SAPHO syndrome is illustrated by Kerrison et al who reported dramatic pain relief in 7 pediatric patients following an initial dose of pamidronate [4]. In keeping with the observation that some children with SAPHO syndrome may lack dermatological features, these authors included three patients with bone lesions only, and who therefore were similar to our CRMO patients. The skin lesions in the remaining patients consisted of psoriasis/pustular psoriasis. The dose of pamidronate varied between a maximum of 30–60 mg/day, given as 3-day cycles. The frequency of pamidronate cycles was determined by symptom recurrence. Five patients had clinical relapse of osteitis, which responded to repeat pamidronate in all patients. The authors did not comment on dermatological response following pamidronate. Finally, complete pain resolution and decrease in adjacent soft tissue swelling was documented by Compeyrot-Lacassagne et al in two pediatric patients with chronic inflammatory lesions of the mandible within one week of pamidronate [28]. Similar to Kerrison's and our series, both of these patients had recurrence of inflammatory osteitis, 6 and 17 months after the initial treatment. CT scan together with clinical features and laboratory measurements was used to monitor disease activity.

Although the rapid pain relief after bisphosphonates in multiple bone disorders is well documented, its etiology remains incompletely understood. Ghilardi et al have studied osteolytic bone lesions in malignancies, and suggest that overactive osteoclasts induce acidic environment, which allows bone resorption by osteoclasts, contributing to the bone destruction and damage to primary afferent fibers [34]. Bisphosphonates neutralize the nociceptive acidic environment rapidly by inactivating osteoclasts. This mechanism could explain the rapid pain relief in our patients. The prompt improvement in soft tissue swelling adjacent to bone lesions, which was observed in several of our CRMO patients, most likely reflects the anti-cytokine effects of pamidronate.

Compared to the above reports on effective analgesic effects of pamidronate in diverse pediatric CNO disorders, our study was unique in that all patients had correlating MR images to document bone inflammation and its resolution at pre-determined time points [4,20]. The advantage of obtaining MRI at baseline and during follow-up is multifold. MRI is extremely sensitive in detecting bone marrow edema and soft tissue edema that are present in inflammatory processes such as CRMO. It is more sensitive indicator of disease activity than the lytic changes seen on plain radiography. It is especially helpful in evaluating lesions that are difficult to visualize by plain radiography, such as spine and pelvis [35]. At baseline, all patients demonstrated bone and/or soft tissue inflammation by MRI. MRI improved remarkably after the first pamidronate treatment, with further resolution with subsequent treatments, supporting the proposed anti-inflammatory role of bisphosphonates [4,27]. Because CRMO can result in pain from non-inflammatory bone changes such as vertebral body compression fractures from spinal CRMO [3], we recommend a repeat MRI assessment in cases of suspected flare, particularly if repeat treatment with pamidronate is contemplated.

In this study we show for the first time data on bone remodeling markers in pediatric CRMO patients. In adult onset SAPHO syndrome, increased levels of serum osteocalcin have been observed in some patients, suggesting greater importance of bone resorption compared with bone formation in this disorder [21]. Although histology reveals increased osteoclasts in early CRMO lesions [8], and early radiographs reveal osteolysis, we were not able to show generalized increase in bone resorption markers or in markers of bone formation compared to age-specific norms. Four patients had baseline uNTX/uCr values above the 75^th ^% percentile for age, but we feel that this increase most likely reflects the on-set of puberty, rather than increase from CRMO related osteolysis. All patients had a decrease in uNTX/uCr following pamidronate, which was expected. However, while it is also expected that uNTX/uCr values gradually begin to increase after bisphosphonates are discontinued, it is interesting that no patient relapsed while his/her bone turnover remained suppressed. It is therefore tempting to speculate that bone and adjacent soft tissue inflammation may require functioning osteoclasts for clinical manifestations. However, further studies are required on the role of osteoclasts and on the potential use of uNTX/uCr in pediatric CRMO.

In long-term studies 20–30% of CRMO patients may develop acne, psoriasis, synovitis, inflammatory bowel disease and/or ankylosing spondylitis [3,5,36]. The low rate of these associations in our patients most likely reflects the well documented lag in presentation of the extra-osseous manifestations, and their true incidence will only become apparent with longer follow-up.

Similar to other reviews of pamidronate in CNO, the only observed side-effect in our study was transient acute phase response with the first pamidronate dose [4]. Osteonecrosis of the jaw (ONJ) has emerged as a potential concern for adult patients treated with bisphosphonates. ONJ is in most cases initiated by tooth removal, and most reported cases have had a history of malignancy [37]. To date, ONJ has not been reported in pediatric patients [23,37]. A comprehensive review by Malmgren et al of all 64 pediatric osteogenesis imperfecta patients in Sweden who were treated with pamidronate from 1991–2005 revealed no cases of ONJ. A total of 38 dental surgical procedures were performed in 22 of these 64 patients at the age of 3.4–31.9 years after 0.03–7.9 years of treatment [37]. However, until more pediatric data is collected, we suggest the following: 1) a thorough dental assessment pre-pamidronate; 2) wisdom tooth extraction pre-pamidronate in adolescent patients if recommended by a dentist; and 3) postponement of dental braces or elective dental extractions for at least 6 months following the final pamidonate treatment.

The "minimum effective dose" and ideal duration of pamidronate treatment for CRMO is not known. In this case series pamidronate was used only when conventional treatment was ineffective, and symptoms were of long standing. A much lower dose of pamidronate was required for MRI documented CRMO resolution following relapse, suggesting that early CRMO may be more amenable to bisphosphonate treatment than persistent disease. Pamidronate was discontinued once MRI abnormalities had resolved. At that time, uNTX/uCr remained suppressed, and ongoing pamidronate treatment would not have likely offered additional benefit. However, our experience as well as that of others [4,28] suggests that many pediatric CRMO/CNO patients who are treated with pamidronate eventually flare. We have restricted repeat treatment to those patients who have MRI confirmed relapse of bone inflammation, and whose flare is unresponsive to NSAIDs.

Finally, pamidronate has been the only intravenous bisphosphonate available for pediatric patients. However, other intravenous bisphosphonates are becoming available for this age group, including zoledronic acid, which, because of its higher relative potency [38], may achieve the same effect as pamidronate with less number of infusions and possibly longer time interval until relapse.

## Conclusion

Persistent CRMO, comprising up to 25% of all pediatric cases [2], continues to be a management challenge due to intractable pain, and risk for permanent bone deformities. In this sub-group of patients, potential advantages of pamidronate include rapidity of symptom control and improvement in quality of life. It is not yet known whether improvement in bone inflammation following pamidronate will result in decreased bone deformities in future. However, although our results are encouraging, they are uncontrolled and observational. There is now a great need for a randomized controlled trial, with participation of many centers, to help define the place of bisphosphonates in the management of pediatric CRMO and other CNO disorders.

## Abbreviations

CRMO: chronic recurrent multifocal osteomyelitis; CNO: chronic non-bacterial osteitis; NSAIDs: non steroidal anti-inflammatory drugs; MRI: Magnetic resonance imaging; uNTX/uCr: urinary cross-linked N-telopeptides of type I collagen related to creatinine; OI: osteogenesis imperfecta; ONJ: osteonecrosis of the jaw; SAPHO syndrome: synovitis, acne, pustulosis, hyperostosis and osteitis syndrome

## Consent

Written informed consent was obtained from the patients/parents for publication of these case reports and any accompanying images. A copy of the written consent is available for review by the Editor-in-Chief of this journal.

## Competing interests

The authors declare that they have no competing interests.

## Authors' contributions

PMHM conceived of the study, and participated in its design and coordination, and drafted the manuscript. XW, DK and WF participated in the design for diagnostic imaging of the patients, and contributed equally to analysis of radiographs and magnetic resonance imaging. ANA contributed to study design and was responsible for data analysis, and JDK contributed to overall study design and helped draft the manuscript. All authors read and approved the final manuscript.
